# Virulence and Immune Response of *Campylobacter jejuni* Strains in Chicken Embryo Model

**DOI:** 10.1007/s00284-026-04768-7

**Published:** 2026-02-20

**Authors:** Paula Fernanda de Sousa Braga, Emília Rezende Vaz, Simone Sommerfeld, Fabiana de Almeida Araújo Santos, Gabriela Ribeiro da Silva, Alessandra Castro Rodrigues, Isabelle Ezequiel Pedrosa, Alessandra Aparecida Medeiros Ronchi, Adriana Freitas Neves, Belchiolina Beatriz Fonseca

**Affiliations:** 1 Postgraduate Program in Veterinary Sciences, Federal University of Uberlândia, Av. Pará, 1720 - Campus Umuarama - Bloco 2 E – CEP 38405-320, Uberlândia, Brazil; 2 Postgraduate Program in Genetics and Biochemistry, Federal University of Uberlândia, R. Acre, 1004 - Umuarama, Uberlândia - MG– CEP 38405-302, Uberlândia, Brazil; 3 Institute of Biotechnology, Molecular Biology Laboratory, Federal University of Catalão, Setor Universitário, Av. Dr. Lamartine Pinto de Avelar, Catalão, 1120, CEP 75704-020 Goiás Brazil

## Abstract

This study aimed to evaluate the virulence and infection response of *Campylobacter jejuni* (CJ) strains isolated from chickens, as well as a standard strain isolated from humans, to understand better the pathogen-host relationship between CJ and chicken embryos (CE) with an active immune system. With its well-developed vascular structures, the CE model offers a valuable method for studying complex biological systems. We assessed embryo mortality, weight, macroscopic and microscopic lesions, bacterial multiplication within the embryo, and macrophage and lymphocyte (T and B cell) counts using flow cytometry. Additionally, histopathological lesions were examined. CJ killed CE at a dose of 3.7 log CFU/CE; however, lower doses (2.5 log CFU/CE) did not result in embryo death but instead caused macroscopic damage along with mild to moderate lesions. Certain strains were also capable of stimulating the immune system; however, this response varied depending on the strain. These findings underscore the importance of studying the virulence, infection dynamics, and immune responses of various CJ strains, highlighting the utility of the CE model for these investigations.

**Supplementary Information:**

The online version contains supplementary material available at 10.1007/s00284-026-04768-7.

## Introduction

*Salmonella* spp. and *Campylobacter* spp. are among the agents most implicated in foodborne infections. *Campylobacter jejuni* (CJ) infections, strongly linked to contaminated retail chicken by several studies [1], are highly prevalent in commercial broiler flocks. A study showed that 95% of birds can be rapidly colonized by *Campylobacter* spp. once exposed to one single infected seeder bird and remain colonized until market age [2]. This pathogen is highly prevalent in chicken ceca (10^9^ CFU/g), usually without causing clinical disease in adult birds [3]. During slaughter, this bacterium is released from the intestines, contaminating the meat and thus posing risk to humans [4].

Although CJ typically causes a self-limiting disease in immunocompetent individuals, infections can lead to severe outcomes in children, the elderly, and immunocompromised patients [5]. Additionally, extra-intestinal manifestations such as polyarthralgia (i.e., reactive arthritis at multiple sites) or Guillain-Barré syndrome (GBS) may occur [6]. To address these gaps, the effort to reduce *Campylobacter* spp. infections in humans is directly linked to a better understanding of the infection mechanisms and host response. However, the molecular basis of CJ’s virulence mechanisms is strain-dependent. For this reason, assessing the phenotypic characteristics of isolated strains is essential to better understand the pathogen-host relationship. While cell culture studies are efficient for characterizing virulence traits and host immune responses, they cannot capture a full, dynamic, organism-level response. Thus, in vivo models are essential for preliminary studies.

Chicken embryos (CE) represent an accessible and powerful in vivo system for studying complex biological processes. They possess well-developed vascular structures, offer high experimental reproducibility, and are inexpensive and easy to handle [7]. The CE model also has a long and distinguished history in developmental biology, and has contributed fundamentally to advances in immunology, genetics, virology, cancer, and cell biology, making it one of the most versatile experimental systems available [8].

Although CJ can kill CE at high doses if it infects CE in the early stages of incubation [9, 10], we know little about the lesions or inflammatory responses this bacterium causes in CE. To our knowledge, no studies have investigated the immune response in CE following CJ infection.

Given the importance of studying the virulence, infection, and immune response of various strains of CJ and the established utility of the CE model, we aim to evaluate the virulence and infection potential of CJ strains isolated from chicken as well as standard strains isolated from humans. This approach will allow us to better understand the pathogen-host relationship between CJ and CE at stages in which embryos possess an active immune system.

## Materials and Methods

For this experiment, we tested a total of 177 eggs (Ross 308 lineage) obtained from a commercial broiler hatchery in the Uberlândia region (MG), Brazil. Of these, 37 eggs were used for the mortality assay, and 140 eggs were used for virulence and infection analyses. The 140 eggs were divided into two groups of 70 eggs each, across two identical experiments conducted at different times (biological replication). Although we had a high number of eggs, it is important to mention that to perform analyses such as cytometry, we need a large amount of fresh blood (collected and processed at the same time) [11, 12]. Since the blood volume in the chicken embryo (CE) is low, and sometimes quality issues arise, such as the presence of fibrin, it was not possible to use the same animals for all analyses. Therefore, we worked with 3 chicken embryos per sample for cytometry analysis and 3 chickens per sample for histopathology or microbiology. For viability analysis and weight, we performed the analyses on all chicken embryos from the first and second repetitions.

The eggs were transported to the incubation laboratory at the Veterinary Medicine Faculty of the Federal University of Uberlândia. Each egg was assigned a unique identification number and incubated in a Premium Ecologica^®^ IP30 incubator, with an average humidity of 55% and a temperature of 38 °C.

### Strains

In this experiment, four strains were used. One of them was strain IAL 2383, isolated from humans which harbors the genes *FlaA*,* pldA*,* CadF*, and *CiaB* [13]. There is no information available regarding the presence or absence of other virulence genes; however, this strain was isolated from an infected human. The other three strains (C030/30, C046/10, and C092/6) were isolated from chickens [14]. Regarding strain 092/6, it harbors all major virulence genes related to motility, adhesion, invasion, protection against oxidative stress, and cellular damage (*FlaA*,* FlaB*,* pldA*,* SodB*,* CadF*,* CiaB*, and *cdtABC*). Strain C046/10 possesses several key virulence genes (*pldA*,* SodB*,* CadF*,* CiaB*, and *cdtABC*); however, it lacks the genes *FlaA* and *FlaB*, which are important for motility and invasion [15]. Regarding strain 030/3, it was previously described and investigated by Peres et al. (2023) [14]. Although the authors informed us that this strain harbors all previously described virulence genes (*FlaA*,* FlaB*,* pldA*,* SodB*,* CadF*,* CiaB*, and *cdtABC*), this information was communicated informally, as the manuscript did not specify which virulence genes were present in each individual strain.

### Previous Test

We first performed a pilot test to investigate the mortality rate of embryos infected by bacterial samples. To optimize study design, the mortality rate needed to be low to ensure an adequate number of embryos for laboratory tests. We used 37 viable eggs, divided into the following groups: (i) a total of five (5) CE inoculated with CJ, strain IAL 2383; (ii, iii, iv) four (4) CE in each group inoculated with CJ, strains C030/30, C046/10, C092/6, respectively; (v) ten CE inoculated with *Salmonella* Typhimurium (ST) isolated from chicken; (vi) ten CE as a negative control inoculated with sterile 0.9% NaCl. We randomly selected the bacterial strains for the experiment from our bank of strains isolated from broiler chickens. For this step, CJ strains were grown in CCDA (charcoal cefoperazone deoxycholate agar) medium (Acumedia^®^) at 40 °C in a micro-aerobic atmosphere for 48 h and ST was grown in nutrient agar at 37 °C for 24 h. On the 10th day of embryonic development, eggs were candled to detect and discard those that were unfertilized or contained dead embryos, then inoculated as described below. A larger number of controls were used to determine the number of CE needed for the next phase of research.

A hole was aseptically made in the eggshell, and 3.7 log CFU/CE of bacterial suspensions, prepared from an overnight culture diluted in sterile saline solution, were inoculated into the allantoic cavity. On the 17th day of incubation, gross lesions and mortality were evaluated, and allantoic fluid was collected for plating on the CCDA (charcoal cefoperazone deoxycholate agar) medium (Acumedia^®^) to count CJ in the fluid.

### Virulence and Infection Test

Due to the high mortality rate in the pilot test, we decided to use a lower dose of CJ and ST (2.5 logs CFU/CE) administered via the allantoic cavity. The experiment was performed twice to obtain biological replicates. A total of 140 eggs were tested for virulence and infection, with 70 eggs per replicate.

On the 10th day of incubation, each egg within groups of 10 was weighed, identified, and inoculated with one of the following agents via the allantoic fluid: ST (positive control), CJ strains IAL 2383, C030/3, C046/10, and C092/6, a probiotic *Bacillus subtilis* (BS) strain as a negative control [16] and sterile saline solution as another negative control.

The BS and ST bacteria were cultured on nutrient agar (Acumedia^®^) medium at 37 °C for 24 h, while CJ’s strains were grown in CCDA medium (Acumedia^®^) at 40 °C in a micro-aerobic atmosphere for 48 h. After the inoculation, egg viability was assessed daily. On the 17th day of incubation, we weighed the CE without the embryonic annexes and analyzed the lesions. We also collected blood, allantoic fluid, and liver tissue samples.

#### Mortality, Multiplication of CJ in the CE Allantoic fluid, Weight Change and Macroscopic Lesions

A precision scale M214-AIH (Bel Engineering^®^) was used to weigh the eggs before inoculation and the embryos on the 17th day of incubation. As the weight of the eggs is not completely uniform, to avoid errors, the final embryo weight was adjusted, assuming a standard egg weight of 50 g using a simple rule of three. Embryo mortality was assessed daily in the dark using a flashlight to determine whether each embryo was active or deceased. Mortality index, weight, and macroscopic lesions were recorded for all tested CE.

The allantoic fluid was aseptically collected using a 5 mL syringe and transferred to a laminar flow hood for processing. In each experimental repetition, we sampled three CE per group to collect a total of 6 samples. We diluted 100 µL of each sample in 900µL of sterile saline solution, performing serial tenfold dilutions for plating. ST was plated in duplicates on nutrient agar (Acumedia^®^) and incubated at 37 °C for 24 h for colony counting. CJ strains were plated on CCDA medium (Acumedia^®^) and incubated at 40 °C in a microaerobic atmosphere for 48 h for colony counting.

#### Macrophage and Lymphocyte Counting by Flow Cytometry Analysis

The whole blood was collected from 17-day-old CE aseptically, using a 5 mL syringe for each embryo, with approximately 2 mL of blood collected into a blood test tube containing 50 µL of EDTA. In each experimental repetition, we randomly sampled three CE per group, totaling six samples. The blood was centrifuged at 200 x g for 5 min at room temperature to separate the blood plasma. The total leukocyte fraction was collected and added to an erythrocyte lysis buffer (BD^®^) and incubated for 15 min at room temperature. The cells were then centrifuged at 200 x g for 5 min at room temperature and incubated with PBS-BSA 5% for 10 min at room temperature. Next, we added 2.5 µL of Mouse Anti-Chicken CD8-FITC (Southern Biotech^®^), CD4 Pe-Cy7 (Southern Biotech^®^), and Monocyte/Macrophage-PE (Southern Biotech^®^) and incubated for 1 h at 4 °C in the dark. The isotypes Mouse IgG1-PE (Southern Biotech^®^), IgG2-FITC (Southern Biotech^®^), and IgG1- Pe-Cy7 (Southern Biotech^®^) were used to perform gate and analysis strategy. The cells were washed twice with a wash buffer and centrifuged for 5 min at 200 x g at room temperature. Next, 100 µl of PBS1x was added, and the cells were analyzed by flow cytometry (Attune-Thermo^®^). Negative control with unlabeled cells was included in each test. Cells were analyzed for at least 1,200,000 events at the lymphocyte gate, and data were analyzed using the software provided by Attune Flow Cytometer (Thermo Fisher Scientific^®^).

#### Histopathology

Liver samples were collected, and the tissues were preserved in a 10% formalin solution for processing. The tissues were dehydrated using graded ethanol concentrations (85%, 95%, and 100%), followed by two immersions in 100% xylene, then embedded in liquid paraffin. The images were analyzed in an optical microscope (Nikon Y-THM^®^) with a magnification of 40x.

Two experienced pathologists analyzed all slides without knowledge of the treatment groups. Then, lesions were identified and scored for severity, and slides from the control group were re-evaluated to confirm normal histology. The control samples were used as a guide for normal histological appearance. All slides were re-examined against the negative control slide to ensure accurate recognition and grading of lesions.

The slides were evaluated semi-quantitatively, using the negative control as a reference, for histological evidence of inflammatory lesions and hemorrhage [17]. The inflammation severity was evaluated based on the percentage of the perivascular inflammatory cell count relative to the total cells in the same area. Briefly, the score system was: 0, no inflammatory cells; 1, inflammatory cell count < 25%; 2, inflammatory cell count between 25 and 50%; 3, inflammatory cell count > 50% [17]. Hemorrhage was evaluated separately from inflammatory infiltrates. Hemorrhage was defined as presence of extravascular erythrocytes in the hepatic parenchyma (distinct from intravascular congestion) and classified semi-quantitatively as 0 = absent; 1 = mild (1–2 small foci or ≤ 5% area); 2 = moderate (> 2 foci or 5–20% area); 3 = severe (> 20% area or diffuse/coalescent hemorrhage). Scoring was performed on 10 non-overlapping fields per slide at 40× (with confirmation at higher magnification) [18].

### Statistical Analyses

The Kolmogorov–Smirnov test was performed to assess whether the data were parametric. For non-parametric data, a square root transformation was applied (in the case of the TCD4 + ratio). We used ANOVA (*p* < 0.05) followed by Tukey’s test for parametric data or the Kruskal-Wallis test for non-parametric data. For association analysis between the number of bacteria and gross lesions, we used the Fisher’s exact test. GraphPad Prism 9.0 software was used, with a 95% confidence interval.

## Results

### Mortality, Multiplication of CJ in the CE Allantoic fluid, Weight Change and Macroscopic Lesions

**Fig. 1:**
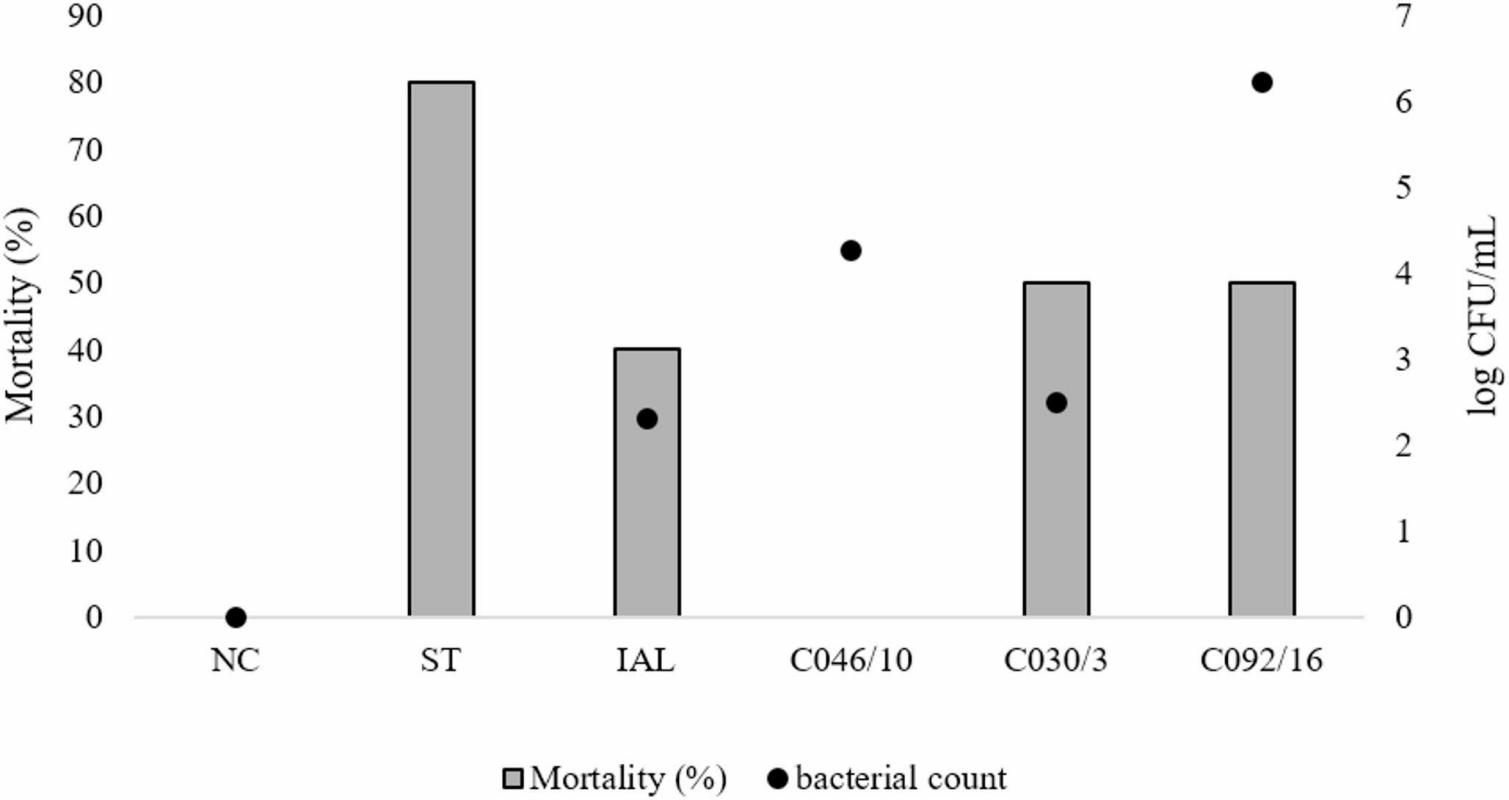
Mortality and bacterial count in chicken embryo at 17th day of incubation inoculated with different strains of CJ at 10 days of incubation. NC: negative control; ST: ST; IAL: CJ strain IAL 2383; C046/10, C030/3, C092/16: CJ strain isolated from chicken. As 90% of the embryos from the positive control (ST) were dead, the bacterial count was not performed in this group. We performed just descriptive statistical analysis

When we inoculated 3.7 log CFU/CE of ST or CJ, there was high mortality in CE. Infection by ST resulted in 80% (8/10) of embryo mortality. The embryo mortality of CJ was 40% (2/5), 50% (2/2), 0% (0/4) and, 50% (2/2) in IAL 2383, C030/3, C046/10, and C092/6, respectively (Fig. 1). The average bacterial counts in the allantoic fluid were 2.30; 4.26; 2.49; and 6.22 log CFU/mL in CJ strains IAL 2383, C030/3, C046/10, and C092/6, respectively.

When inoculated with a dose of 2.5 log CFU/CE, the mortality caused by CJ was low (Table 1). Only one CE died in the strain (C030/3) and ST (PC), indicating that a low infective dose did not lead to high embryonic mortality (Table 1).

**Table 1:** Mortality (%) and gross lesions in the different groups

		NC	BS	ST	IAL	C046/10	C030/3	C092/6
	Mortality (%)	0.0 (0/20)	0.0 (0/20)	5.0 (1/20)	0.0 (0/20)	0.0 (0/20)	5.0 (1/20)	0.0 (0/20)
	**Urate increase**	0.0 (0/20)	0.0 (0/20)	10.5 (2/19)	10.0 (2/20)	15.0 (3/20)	10.5 (2/19)	5.0 (1/20)
**Lesions (%)**	**Liver enlargement**	0.0 (0/20)	0.0 (0/20)	5.3 (1/19)	0.0 (0/20)	0.0 (0/20)	0.0 (0/19)	0.0 (0/20)
	**Greenish liver**	0.0 (0/20)	0.0 (0/20)	10.5 (2/19)	0.0 (0/20)	0.0 (0/20)	0.0 (0/19)	0.0 (0/20)
	**Milky allantoid**	0.0 (0/20)	0.0 (0/20)	21 (4/19)	35 (7/20)	15 (3/20)	10.5 (2/19)	5.0 (1/20)
	**Small embryo**	0.0 (0/20)	0.0 (0/20)	5.3 (1/19)	0.0 (0/20)	0.0 (0/20)	0.0 (0/19)	0.0 (0/20)
	**Enlarged spleen**	0.0 (0/20)	0.0 (0/20)	0.0 (0/19)	10.0 (2/20)	5.0 (1/20)	5.3 (1/19)	0.0 (0/20)

NC: negative control; ST: Chicken embryo (CE) inoculated with *Salmonella* Typhimurium (ST); IAL: CE inoculated with *Campylobacter jejuni* (CJ) strain IAL 2383; C046/10, C030/3, C092/16: CE inoculated with *Campylobacter jejuni* (CJ) strain C046/10, C030/3, C092/16 isolated from chicken. BS: *Bacillus subtilis* (probiotic strain). We performed just descriptive statistical analysis

Regarding the lesions, milky allantoid and increased urate were the most observed lesions in CE inoculated with CJ. CE infected by CJ didn’t present a greenish or enlarged liver (Table 1). However, an enlarged spleen occurred in two CE inoculated with IAL 2383, and one inoculated with CJ C046/10, and C030/3 (Table 1).

**Fig. 2:**
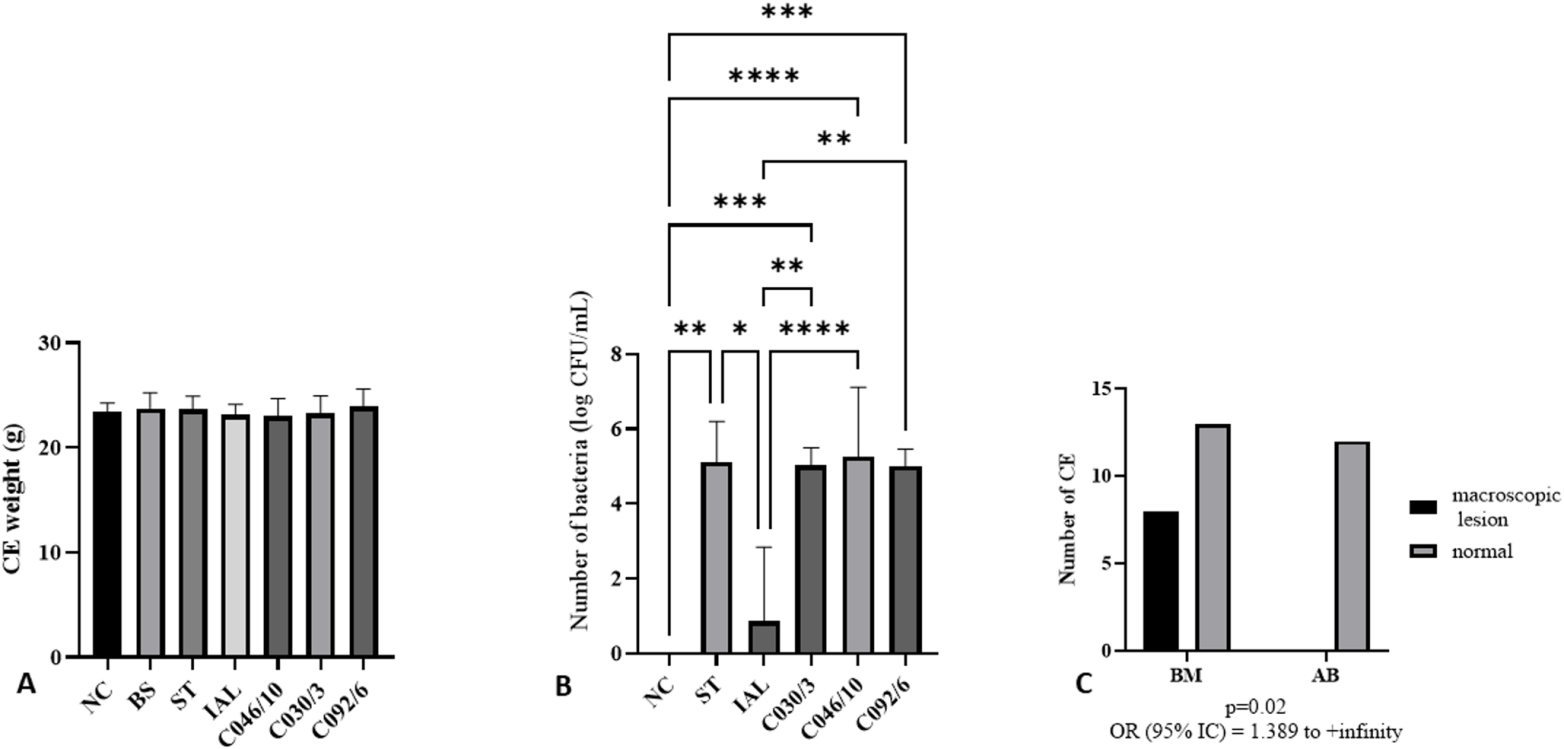
Embryo mean weight, bacterial count in allantoic fluid, and the association between bacterial count and gross lesion. A: Embryo weight in different groups; B: Bacterial count in different groups; C. Association between bacteria multiplication and gross lesion in different groups. We used ANOVA and turkey test (**A**), Kruskal-Wallis test (**B**) or Fisher test (**C**). Statistical differences were considered when *p* ≤ 0,05 (no asterisk: *p* > 0.05; *: *p* ≤ 0.05, **: *p* ≤ 0.01, ***: *p* ≤ 0.001). NC: negative control; ST: CE inoculated with *Salmonella* Typhimurium (ST); IAL: Group of CE inoculated with *Campylobacter jejuni* (CJ) strain IAL 2383; C046/10, C030/3, C092/16: CE inoculated with CJ strain C046/10, C030/3, C092/16 isolated from chicken. CE: chicken embryo; BM: bacteria multiplication; AB: absence of bacteria, OR: odds ratio; IC: confidence interval

CJ as well as ST, did not lead to a decrease in body weight (Fig. 2A). Like ST, all other strains multiplied in the allantoic fluid in the same amount, except for CJ IAL 2383, whose count was lower than the others (Fig. 2B). Bacteria were quantified in all embryos inoculated with CJ, except for CJ IAL 2383, where only 1 CE out of 6 tested had measurable bacteria. In the only embryo where IAL replicated, the bacterial count was 5.17 log CFU/ml.

We analyzed the association between the number of bacteria in allantoic fluid and gross lesions in CE, considering all groups. The results show an association between the absence of bacteria and the absence of lesions (Fig. 2C), with an odds ratio (OR) ranging from 1.389 to infinity (Fig. 2C).

### CJ Does not Cause an Increase of Blood Monocytes and Lymphocytes CD8 + but some Strains Can Increase Lymphocytes CD4 + after 7 Days of Inoculation

**Fig. 3:**
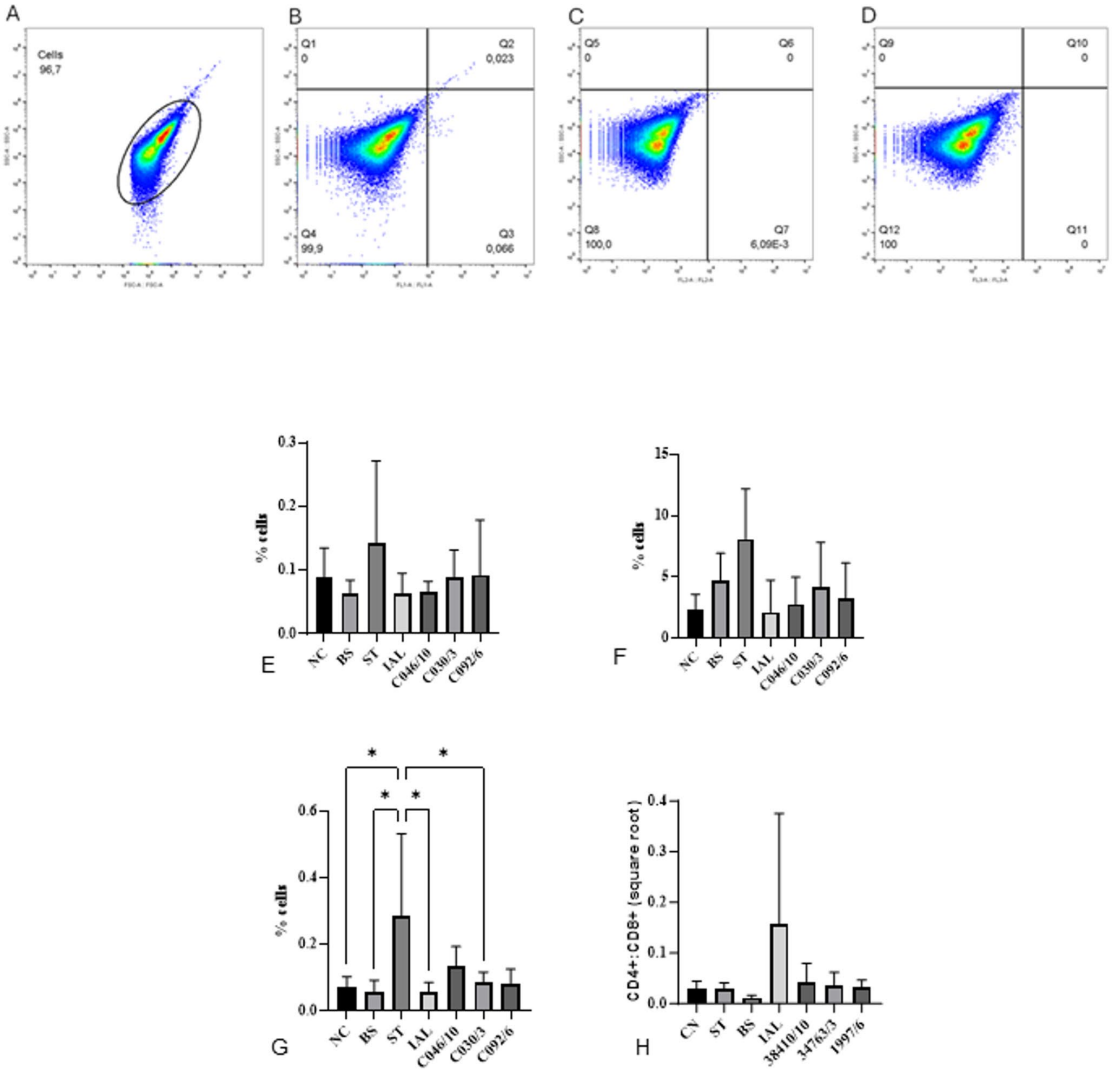
Example of the gating strategy for chicken embryo blood and mean of Monocytes, Lymphocytes CD8 + and CD4+, and Lymphocyte CD4+:CD8 + ratio in CE blood inoculated with different strains of CJ 7 days after the inoculation. (**A**) y axes: SSC-A, x axes: FSC-A, the egg-shaped gate represents the total leukocytes gate; (**B**) y axes: SSC-A, x axes: CD8-FITC; (**C**) y axes: SSC-A, x axes: Monocyte/Macrophage-PE; (**D**) y axes: SSC-A, x axes: CD4-PE-Cy7. Data represents one biological replicate. A total of 1.200.000 cells were recorded on a Thermo Accuri II flow cytometer. (**E**) Monocytes; (**F**) Lymphocyte CD8+; (**G**) Lymphocyte CD4+. (**H**) TCD4+:TCD8 + ratio. We used ANOVA and Tukey test. The data of the CD4+:CD8 + ratio was not parametric, so we normalized the data using a square rate. * Statistical differences were considered when *p* ≤ 0,05 (no asterisk: *p* > 0.05; *: *p* ≤ 0.05, **: *p* ≤ 0.01, ***: *p* ≤ 0.001). NC: negative control; ST: Chicken embryo (CE) inoculated with *Salmonella* Typhimurium (ST); IAL: CE inoculated with *Campylobacter jejuni* (CJ) strain IAL 2383; C046/10, C030/3, C092/16: CE inoculates with CJ strain C046/10, C030/3, C092/16 isolated from chicken

The results of the isotype controls allowed us to minimize the issue related to nonspecific binding of the antibodies used (Supplementary Fig. 1). The gating strategy for CE blood is shown in Fig. 3A, representing the total leukocyte gate. The CD8-FITC graph, Monocyte/Macrophage-PE, and CD4 PE-Cy7 graphs are shown in Fig. 3B, C, and D, respectively. Data represents one biological replicate. A total of 1,200,000 cells were recorded using a Thermo Accuri II flow cytometer.

We found a higher percentage of TCD8 + cells in the blood of CE when compared to TCD4 + cells (*p* < 0.05). We noticed that the percentage of monocytes and TCD8 + lymphocytes did not change in either negative or treated groups (Fig. 3E and F). For CD4 + T cells, CE infected with CJ strains C046/10 and C092/6 showed no statistically significant differences from the positive control (ST) and were similar to the negative control and group infected by a probiotic strain (BS) (Fig. 3G).

We analyzed the lymphocyte CD4+:CD8 + ratio, and although without a statistical difference, the strain IAL 2383 showed a standard deviation of CD4+:CD8+ (Fig. 3H).

### Some Strains of CJ Can Induce Discrete or Moderate Inflammatory Changes in CE

**Fig. 4:**
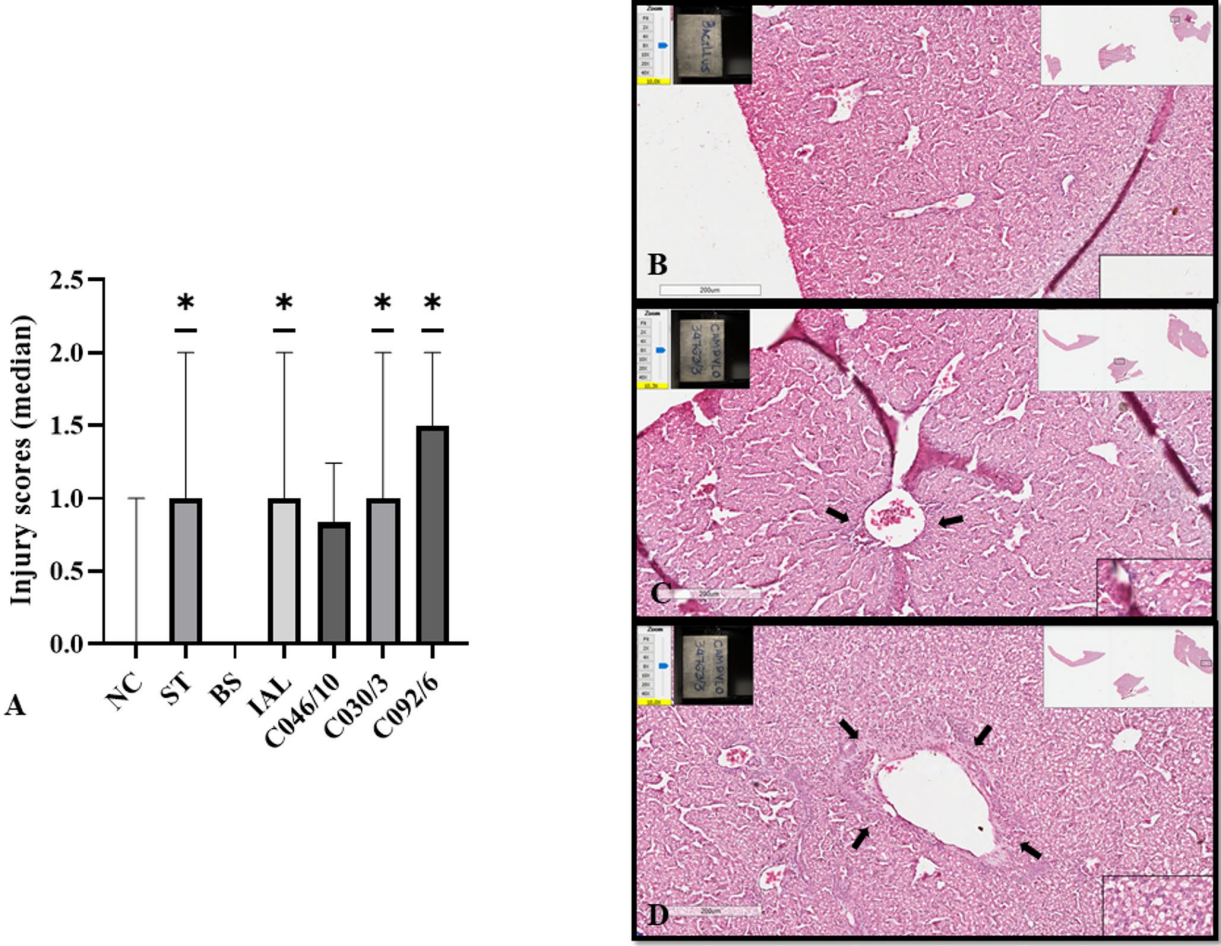
Median of scores of lymphocytic infiltrates in liver of CE inoculated with different strains of CJ. (**A**) Median of lymphocytic infiltrates scores in the liver of CE inoculated at 10 days of incubation and evaluated at 17 days of incubation. (**B**) Score zero (normal score). **C**: discrete inflammatory score. **D**. moderate inflammatory score. The arrow shows perivascular lymphocytic infiltrates. Statistical differences were considered when *p* ≤ 0,05 (no asterisk: *p* > 0.05; *: *p* ≤ 0.05). NC: negative control; ST: Chicken embryos (**CE**) inoculated with *Salmonella* Typhimurium (ST); IAL: CE inoculated with CJ strain IAL 2383; C046/10, C030/3, C092/16: CE inoculates with CJ strain C046/10, C030/3, C092/16 isolated from chicken. PS: It is normal to find infiltrates of perivascular lymphocytes in birds, then we classified the infiltrates by score using the Wilcox test

As it is normal to find granulocytes around portal spaces in birds, we facilitate the analysis by evaluating the extent of the granulocytes around portal spaces using scores: zero (typical score) (Fig. 4B), discrete inflammatory score (Fig. 4C) and moderate inflammatory score (Fig. 4D). The strains CJ IAL, C030/3, and C092/6 induced inflammatory changes in the livers of CE similar to those in the positive control (ST). The observed scores were discrete or moderate (Fig. 4A). No significant differences in inflammatory changes were observed in CE inoculated with NC, BS, or CJ046/10 (Fig. 4A).

## Discussion

Understanding how *Campylobacter jejuni* behaves in the CE is essential for defining both its pathogenic mechanisms and the suitability of CE as an experimental model. Although CE has been used to assess virulence in several avian pathogens [19], data regarding CJ infection dynamics and its effects on embryonic development remain limited.

To investigate CJ pathogenicity in CE, we performed a pilot experiment to assess embryonic susceptibility. Our results demonstrated that mortality was both strain- and dose-dependent, with IAL, C030/3, and C092/16 inducing moderate to high mortality, while the positive control ST caused the highest mortality, consistent with previous reports [19]. It is known that *Campylobacter* can kill CE early [10] or by 10 days of incubation with a high infective dose [20, 21], and embryonic death can serve as an essential tool for assessing the virulence of the strains. Interestingly, only certain CJ strains (C030/3 and C092/6) showed detectable multiplication in the allantoic fluid, whereas others (IAL 2383 and C046/10) did not, despite causing lesions or death at higher doses. These observations suggest that embryonic mortality is not strictly correlated with bacterial replication, highlighting the potential involvement of early host-mediated responses or strain-specific virulence factors.

To explore sublethal effects, we conducted a low-dose experiment (2.5 log CFU/CE) aimed at reducing mortality while assessing organ lesions, inflammation, and immune responses. Differences in mortality between the pilot and low-dose experiments indicate that lethality emerges only when a threshold of virulence and bacterial burden is reached, supporting the strain-dependent nature of CJ pathogenicity [22]. A key and unexpected finding was the decoupling between bacterial detection and pathogenic effects. IAL2383 consistently induced mortality in the pilot test yet replicated poorly in the allantoic cavity under low-dose conditions. This suggests that CJ-induced damage does not require large-scale extracellular proliferation. Therefore, bacterial quantification in the allantoic fluid alone fails to predict disease outcomes, an important caveat for model validation.

Gross lesions (milky allantoic fluid, urate deposition, splenomegaly) and hepatic inflammation were consistently observed across CJ-infected embryos, including those without detectable bacterial loads. This indicates the participation of host-driven mechanisms of injury likely triggered by early bacterial cues [23–26]. These findings contrast with studies describing CJ as non-invasive or lesion-silent *in ovo*, suggesting that pathogenicity has been underestimated when using only high-mortality endpoints [22]. CE therefore enables the study of sublethal, organ-specific consequences of CJ infection.

Our analysis of circulating monocytes revealed no differences among groups, and their overall proportion was consistent with values previously reported for embryos at this developmental stage [7]. Given the short residence time of monocytes in the bloodstream before migration into tissues, it is likely that any early monocytic response to CJ occurred before the sampling point (7 dpi), resulting in a normalized circulating profile [27]. This interpretation aligns with the histopathological evidence of hepatic inflammation, suggesting that monocyte recruitment may have shifted rapidly to infected tissues rather than remaining detectable in peripheral blood. Therefore, the lack of differences in monocyte percentages should not be interpreted as absence of participation in the immune response, but rather as a limitation inherent to the sampling window and to the dynamics of embryonic myeloid cell trafficking.

Lymphocyte subpopulations are commonly assessed in avian blood as indicators of immunocompetence [28]. In our study, TCD8 + cells represented a substantial proportion of circulating lymphocytes (2.29–8.09%) (Fig. 3F), exceeding the frequency of TCD4 + cells (Fig. 3G). Although reports describing CD8 + cells in CE blood are lacking, high numbers of TCD8 + lymphocytes have been documented in embryonic bone marrow [29] and CD8αα + T cells are more abundant in embryonic spleen and bone marrow than in post-hatch chickens [30]. This elevated baseline may reflect early ontogenetic events, including the expression of CD8 on most chicken TCR γδ + cells and a CD8 + TCR0 population proposed to migrate from peripheral sites to the thymus during early development [31]. While many bacterial species can stimulate TCD8 + responses [32] our results do not indicate differential activation of this population in CE infected with CJ, ST, or BS. All groups exhibited a TCD8 + profile comparable to the negative control, contrasting with findings in mice, where CD8 + and CD4 + T cells contribute to protection against CJ, with a predominant CD8 + role [33] and with observations in broilers showing increased CD8 + cells in cecal tonsils after CJ challenge [34]. Given that sampling occurred seven days post-inoculation, it is plausible that TCD8 + lymphocytes had already migrated to tissues or reached a steady-state level in circulation.

TCD4 + cells were less abundant than TCD8 + cells across all groups, ranging from 0.072% in the negative control to 0.283% in the positive control (Fig. 3G). Only the positive control (ST) exhibited a clear increase in TCD4+. Embryos infected with CJ strains C046/10 and C092/6 showed TCD4 + levels similar to the negative control and to the probiotic group, suggesting limited systemic expansion of this subset. The absence of elevated TCD4 + levels in strains that produced macroscopic lesions (IAL 2383 and CE30/30) may indicate that TCD4 + lymphocytes had already migrated to tissues, consistent with the expected developmental maturation of the embryonic immune system [31].

The TCD4 + response to specific strains further reflects their pathogenic profiles. CE inoculated with CJ C046/10 displayed TCD4 + frequencies comparable to the positive control, which may indicate lower pathogenicity and a more effective immune response. This interpretation is supported by the absence of mortality in the pilot test (Fig. 1) and the lack of enhanced hepatic inflammation compared with the negative control (Fig. 4A). Conversely, CE inoculated with C092/6 showed a TCD4 + profile similar to the positive control despite evidence of bacterial multiplication, mortality, gross lesions (Table 1) and moderate inflammation (Fig. 4), suggesting greater pathogenicity of this strain. Although the CD4+:CD8 + ratio is commonly used as an indicator of immune competence in many species [24, 27, 35], embryos naturally exhibit a predominance of TCD8 + over TCD4 + cells because full immunocompetence is achieved only after hatch [36–38].

Taken together, CD4 + and CD8 + lymphocyte frequencies alone do not fully explain the CE immune response to CJ seven days post-infection. Considering that (i) CJ IAL 2383 is pathogenic to humans [13], (ii) induces CE mortality at high doses (Fig. 1), (iii) was undetectable in the allantoic fluid of most infected embryos, and (iv) produced clear macroscopic and microscopic lesions (Table 1; Fig. 4), it is likely that this strain elicited an immune response. However, the seven-day sampling interval may have been insufficient to capture transient lymphocyte dynamics in circulation.

In conclusion, low-dose CJ inoculation induced detectable lesions in CE, and the immunological responses varied among strains, reinforcing the strain-dependent dynamics of the host–pathogen interaction. Although the immune stimulation observed at seven days post-inoculation did not follow a uniform response profile, the overall patterns indicate that CE can manifest measurable pathological and immunological outcomes following CJ exposure. Taken together, these findings support the suitability of the CE as a complementary model for studying CJ pathogenicity and host responses, while also underscoring the need for strain-specific analyses in future investigations.

## Supplementary Information

Below is the link to the electronic supplementary material.

Supplementary Material 1
