# Supplementary material for: Virulence and Immune Response of Campylobacter jejuni Strains in Chicken Embryo Model
Source: Curr Microbiol. 2026 Feb 20;83(4):198. doi: 10.1007/s00284-026-04768-7 (PMC12923503; doi:10.1007/s00284-026-04768-7)
Supplement: Supplementary file 1 — Supplementary Material 1 [file 284_2026_4768_MOESM1_ESM.docx]

IgG1-Pe-Cy7

IgG1-PE

IgG2-FITC


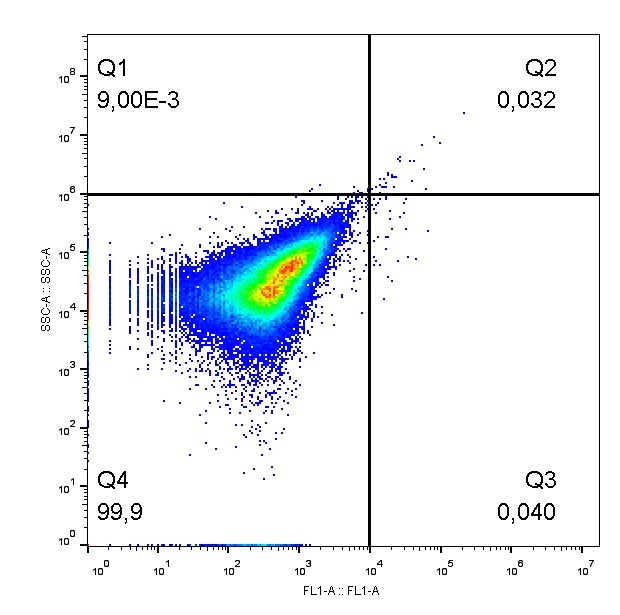

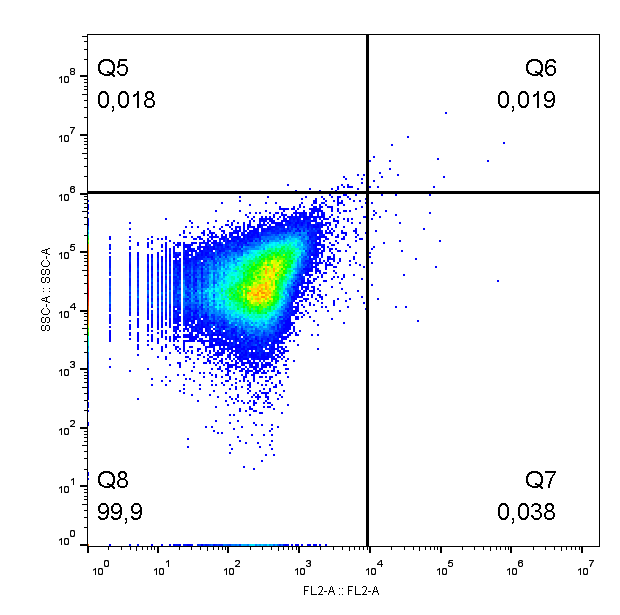

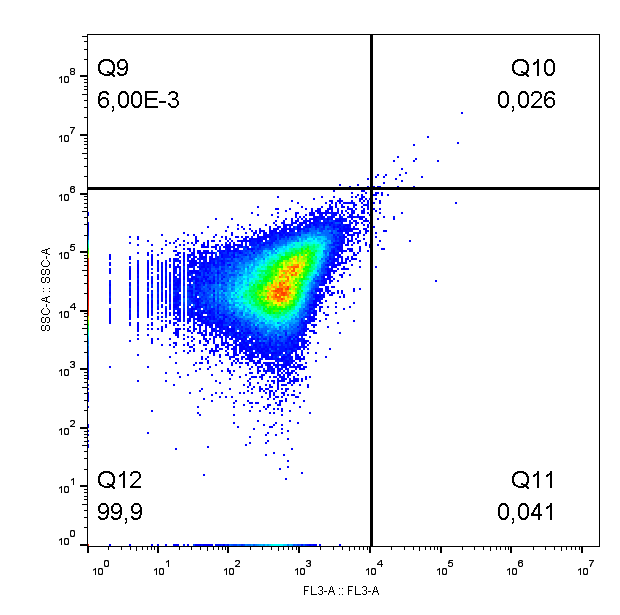

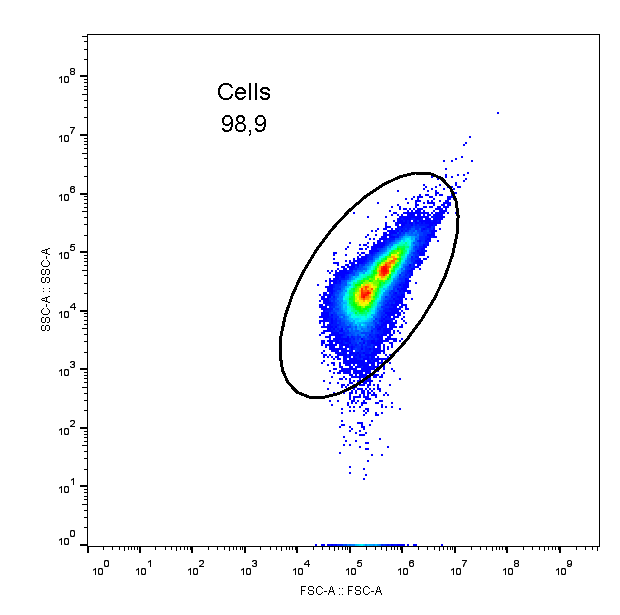


A

B

C

D

**Supplementary Fig.1**

S1: Representative sample of Gates' strategy and use of control isotypes. Isotype IgG2-FITC – FL1 (A), Isotype IgG1-PE – FL2 (B), and Isotype IgG1-PeCy7 – FL3 (C).
